# Kumiss Kefir

**Published:** 1885-05

**Authors:** 


### Kumiss Kefir.

The preparations of Kumiss have never received in this country the degree of attention to which they are fairly entitled. Their undeniable usefulness in affections against which the resources of the practitioner are lamentably deficient, if not absolutely powerless, enhance their claim to a thorough clinical trial. The remedy comes to us with the two-fold credentials of professional recommendation and of a long-enjoyed and tested popularity. Its exhibition in the case of the late President Garfield and subsequent discussion in both the secular and medical press has, if not removed the strange stigma resting on it, at least brought it before the profession more definitely than ever before.

In the *Pharmaceutische Rundschau*, Jan., 1885, are published some interesting points regarding the history, preparation and properties of kumiss and the recently introduced kefir kumiss.

Kumiss is milk after and during the process of fermentation, and was first medicinally employed by the inhabitants of southern Russia. The Tartars prepare kumiss from ass's milk by the addition of blood, which facilitates fermentation, and preserve it in leather vessels. Others again use mare's milk, to which beer-yeast is added. Exposed to a temperature of 68 deg. F. the sugar of the milk is gradually decomposed and a substance entirely different from milk, namely, kumiss, results. It soon became known all throughout Russia, that the nomadic tribes of the south, however reduced they got, through malnutrition and exposure during the winter, could easily regain flesh and strength by the persistent use of kumiss during the summer. Thus the remedy soon gained an excellent reputation in phthisical and other wasting diseases, where the low state of the digestive apparatus and general adynamia called for a readily digestible and at the same time highly nutritious diet. The positively remakable success, which the use of kumiss achieved, especially in phthisis, is of course not due to any influence the remedy exercises over the tissue-changes going on in the lungs, but to the improvement it produces in the nutritive processes of the economy. Rivalling and possibly excelling in ready digestibility all other articles of diet, kumiss heightens the tone of the alimentary tract primarily, improves nutrition and thus to a certain extent is capable of indirectly checking extensive tissue-waste. With increased powers of resistance of the economy, the febrile symptoms naturally abate, and dyspnœa, cough and expectoration decrease. The question, why in the preparation of kumiss cow's milk be not employed in preference to ass's or mare's milk, is easily answered by the fact, that mare's milk, except woman's milk, is more nutritive and more readily digestible, and hence for the preparation of kumiss more eligible than any other.

It is only within the last few years that the discovery of a new ferment, called *Kefir*, has led to the use of cow's milk in the preparation of kumiss. The quality of the cow's milk is of paramount importance in making kefir kumiss. The milk secreted during the first few days following parturition, the so-called colostrum, being little nutritive, but very thin and very easily digestible, is most eligible at the commencement of the kumiss regimen, while gradually the more consistent forms are to be substituted. Next to the quality, the age of the milk claims consideration, the milk just obtained from the cow being fittest for use.

Kefir consists of small hard lumps, of a mushroom-like odor and light-yellow color, resembling the inferior qualities of gum arabic. After being soaked in water kefir swells considerably, grows white and elastic and can be easily torn. Milk appears to be the most favorable soil for its development; when immersed in it, the lumps grow gradually larger, then divide and subdivide, the single parts again growing larger. At first the lumps sink to the bottom but soon begin to rise to the surface raised by small bubbles of carbonic acid attached to them, and remain on the surface all the greater part of the sugar of milk has been changed into carbonic acid, alcohol and lactic acid. The presence of alcoholic fermentation makes itself distinctly perceptible to the ear by the bursting of small bubbles of carbonic acid.

Kern regards the kefir-ferment as consisting of yeast-cells (saccharomyces cerevisiæ, Meyer) and bacillus-like bacteria (dispora caucasica). Kefir resists unfavorable conditions of life to a singular degree; thus it remains intact for two months in a concentrated solution of picric acid or in a 3% solution of chromic acid. To prepare kefir kumiss for actual use, about 7 dr. of kefir are mixed with two glasses of skimmed milk and allowed to stand over night. In the morning the milk is decanted, then diluted with skimmed milk and stored away in sealed bottles; twenty-four hours after, during which period the substance is subjected to repeated shaking, kefir kumiss is obtained ready for use. The following table illustrates its composition as compared with milk and ordinary kumiss:

| In 1000 parts | Milk. | Kefir. | Kumiss. |
|---|---|---|---|
| Albumen | 48 | 38 | 11 |
| Fats | 38 | 20 | 20 |
| Lactose | 41 | 20 | 22 |
| Lactic acid | | 9 | 11 |
| Alcohol | | 8 | 16 |
| Water, salts | 873 | 904 | 918 |

Kefir is much more palatable than kumiss and does not create any digestive troubles like the latter, besides it is much cheaper, as the 2 dr. originally needed (which cost about $1.50), can be used for the preparation of very large quantities of kefir kumiss on account of its extensive propagation.

Kefir kumis is an excellent article of diet in all cases of low vitality, and when associated with iron, a reliable tonic is chlorosis, anæmia and nearly all affections of the respiratory apparatus, but chiefly in incipient phthisis; it is also very valuable in chronic gastric and enteric affections. Kefir is to be taken in the morning, on an empty stomach, and to be drunk slowly, beginning with two glasses, and gradually increaslng the quantity to ten glasses. Mandowski reports very gratifying results from its use in dyspepsia, vomiting from gastric irritation, gactralgia and gastric catarrh, and even in catarrhal pneumonia, phthisis, cancer and syphilis. The numerous kefir hospitals in Russia in the hands of the regular profession can only be considered as an additional favorable testimony to the value of the new remedy.—*Therapeutic Gazette*

